# The Half-Life-Extended IL21 can Be Combined With Multiple Checkpoint Inhibitors for Tumor Immunotherapy

**DOI:** 10.3389/fcell.2021.779865

**Published:** 2021-11-15

**Authors:** Shaoxian Wu, Runzi Sun, Bo Tan, Bendong Chen, Wenyan Zhou, David Shihong Gao, Joshua Zhong, Hao Huang, Jingting Jiang, Binfeng Lu

**Affiliations:** ^1^ Department of Tumor Biological Treatment, The Third Affiliated Hospital of Soochow University, Changzhou, China; ^2^ Department of Immunology, University of Pittsburgh School of Medicine, Pittsburgh, PA, United States

**Keywords:** interleukin 21, checkpoint inhibitors, tumor microenvironment, immunotherapy, mechanisms

## Abstract

In the era of immune checkpoint blockade cancer therapy, cytokines have become an attractive immune therapeutics to increase response rates. Interleukin 21 (IL21) as a single agent has been evaluated for cancer treatment with good clinical efficacy. However, the clinical application of IL21 is limited by a short half-life and concern about potential immune suppressive effect on dendritic cells. Here, we examined the antitumor function of a half-life extended IL21 alone and in combination with PD-1 blockade using preclinical mouse tumor models. We also determined the immune mechanisms of combination therapy. We found that combination therapy additively inhibited the growth of mouse tumors by increasing the effector function of type 1 lymphocytes. Combination therapy also increased the fraction of type 1 dendritic cells (DC1s) and M1 macrophages in the tumor microenvironment (TME). However, combination therapy also induced immune regulatory mechanisms, including the checkpoint molecules Tim-3, Lag-3, and CD39, as well as myeloid derived suppressor cells (MDSC). This study reveals the mechanisms of IL21/PD-1 cooperation and shed light on rational design of novel combination cancer immunotherapy.

## Introduction

Recently, immune checkpoint blockade (ICB) has showed therapeutic efficacy and greatly prolonged survival in cancer patients. However, the response rates of ICB treatment are low for most carcinomas and new approach is needed to further improve cancer immune therapy (Chae et al., 2018; Li et al., 2018). ICB therapy has removed a major roadblock of cancer treatment by targeting molecules that hinder T cell-mediated immune responses (Hodi et al., 2010; Brahmer et al., 2012; Topalian et al., 2012). This new development has ushered in rich opportunities for using immune agonists as combination therapies. Cytokines drive T cell-mediated immune responses by enhancing proliferation, promoting type 1 differentiation, increasing the effector function, and directing the memory generation (Shourian et al., 2019; Zander et al., 2019; Xue et al., 2021). In contrast, molecules such as PD-1 impose “brakes” to an adaptive immune response. Therefore, the cytokine-based immunotherapy is in theory in concert with ICB therapy and promises to further improve clinical response rates.

Interleukin 21 (IL21) is an immune agonist that is an attractive cancer immunotherapeutic (Ma et al., 2003; Ugai et al., 2003; Di Carlo et al., 2004; Sivakumar et al., 2004; Spolski and Leonard, 2008; Xu et al., 2015; Lewis et al., 2017; Deng et al., 2020). Administration of IL21 *in vivo* directly increases the expression of effector molecules on CD8^+^T and NK cell, including granzyme B, perforin and IFN-γ (Kasaian et al., 2002; Brady et al., 2004; Zeng et al., 2005; White et al., 2007). Recently, IL21 was shown to promote the generation of memory stem CD8^+^T cells, thereby should promote a sustained antitumor immune response (Zhang et al., 2005; Klebanoff et al., 2011; Wölfl et al., 2011; Chen et al., 2018). IL21 also synergizes with IL-15 or IL-7 *in vitro* to promote the proliferation of central memory CD8^+^ T cells (Kasaian et al., 2002; Brady et al., 2004; Zeng et al., 2005). Other studies show that Th17 cells produce significantly higher levels of IL21 than either Th1 or Th2 cells and that IL21 is required for the generation of Th17 cells (Korn et al., 2007; Nurieva et al., 2007; Zhou et al., 2007). Th17 cells afford strong antitumor activities by stimulating CD8^+^T cells (Martin-Orozco et al., 2009). In addition to its direct effect on conventional T (T_conv_) cells, IL21 inhibits the suppressive function of regulatory T cells (Tregs) and disrupts their homeostasis (Peluso et al., 2007; Clough et al., 2008; Attridge et al., 2012; Van Belle et al., 2012). Whether IL21 induces other immune regulatory pathways remain to be investigated. All in all, the existing data shows that IL21 strongly promotes the anti-tumor immune response. Indeed, administration of IL21 has shown strong antitumor efficacy in multiple preclinical mouse tumor models (Spolski and Leonard, 2008). Recent preclinical studies showed that recombinant IL21 synergizes with CTLA-4 and PD-1 blockade to inhibit cancer. These results validate the ability of IL21 to be combined with current ICB therapies (Lewis et al., 2017). Since many new checkpoint inhibitors are being evaluated in the clinics, it remains to be studied whether IL21 can be further combined with additional immune checkpoint inhibitors, such as anti-Lag-3 and anti-Tim-3 monoclonal antibodies (mAbs).

Recombinant IL21 has been tested as an antitumor agent in various clinical trials (Davis et al., 2007; Thompson et al., 2008; Davis et al., 2009; Schmidt et al., 2010; Petrella et al., 2012; Bhatia et al., 2014). The clinical program has advanced to phase II with promising antitumor activities and acceptable toxicity. However, the short half-life of IL21 reduces the *in vivo* levels of IL21 and requires frequent dosing, which limits its clinical application. In this study we first examined the therapeutic efficacy of a half-life-improved IL21 in preclinical mouse tumor models. We then investigated the immune mechanisms of combination therapy. Next, we determined whether IL21 induced the expression of additional immune checkpoint molecules, such as Lag-3, Tim-3, and CD39. Lastly, we examined whether targeting these molecules in triple and quadruple combinations therapies would further increase therapeutic efficacy.

## Results

### IL21R Was Differentially Expressed Among Tumor-Infiltrating Immune Cells

**FIGURE 1:**
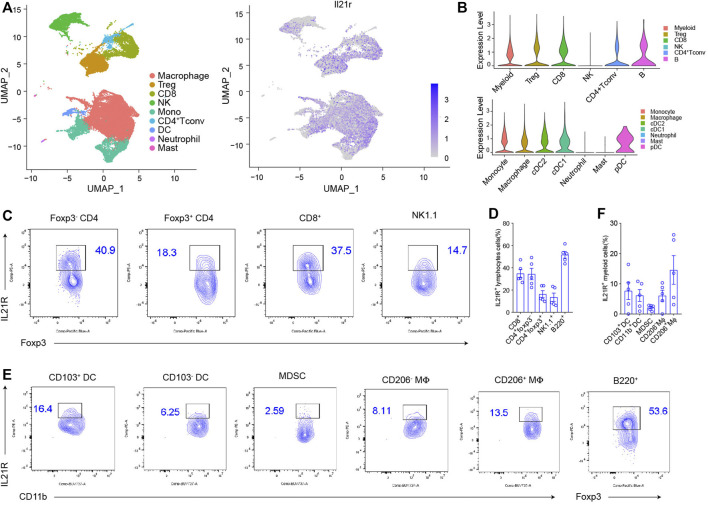
IL21R was differentially expressed among tumor-infiltrating immune cells **(A)** Cluster analysis. Immune cells from vaccinated MC38 mice were drawn by unified manifold approximation and projection (UMAP) dimensionality reduction of scRNA-seq data, and were colored by the expression of IL21R. **(B)** Violin plot shows the expression of IL21R in different immune cell subsets. Each dot represents a single cell. **(C)** Representative flow cytometry scatter plots of IL21R expression on tumor-infiltrating lymphocytes. (Foxp3^-^CD4^+^: CD45^+^CD4^+^Foxp3^-^, Foxp3^+^CD4^+^: CD45^+^CD4^+^Foxp3^+^, CD8^+^: CD45^+^CD8^+^, NK cell: CD45^+^CD8^-^CD4^-^NK1.1^+^, B cells: CD45^+^CD8^-^CD4^-^B220^+^). **(D)** Quantitative percentage of IL21R in various immune cells. **(E)** Representative flow cytometry analyses of IL21R expression on tumor-infiltrating myeloid cells (CD103^+^DC: CD45^+^Gr1^-^MHCII^+^CD24^+^CD103^+^, CD103^-^DC: CD45^+^Gr1^-^MHCII^+^CD24^+^CD103^-^CD11b^+^, MDSC: CD45^+^CD11b^+^Gr1^+^, CD206^-^Mφ: CD45^+^Gr1^-^MHCII^+^F4/80^+^CD206^-^Arg-1^-^, CD206+Mφ: CD45^+^Gr1^-^MHCII^+^F4/80^+^CD206^+^Arg-1^+^). **(F)** Quantitative percentage of IL21R in various immune cells.

The cellular response to IL21-based therapy is dependent on the expression of its receptor IL21R. We examined IL21R expression on immune cells in the tumor microenvironment (TME). First, we analyzed published single-cell RNA-sequencing (scRNA-seq) data of mouse MC38 tumors (Figures 1A,B) (Zhang et al., 2020). IL21R was highly expressed on a broad range of tumor-infiltrating immune cells, including CD4^+^ T_conv_ cells, T regulatory cells (Tregs), CD8^+^ T cells, B cells, NK cells, macrophages, monocytes, and dendritic cells (DCs) but minimally expressed in neutrophils and mast cells (Figures 1C,D). We confirmed IL21R expression at the protein level by analyzing MC38 tumors with flow cytometry. IL21R was expressed on 41% of CD4^+^ Foxp3^-^ T cells, 18% of Tregs, 38% of CD8^+^ T cells, 15% of NK cells, and 54% of B cells (Figures 1C,D and Supplementary Figure S1A). IL21R was also expressed on a smaller fraction of myeloid cells (16% of type 1 DCs (DC1s), 6% of type 2 DCs (DC2s), 3% of myeloid-derived suppressor cells (MDSCs), 8% of M1 macrophages, and 14% of M2 macrophages (Figures 1E,F). Interestingly, we observed that IL21R was expressed on more than 79% of T and B lymphocytes in lymph nodes and spleen (Supplementary Figures S1B,C). These data show that IL21 can directly act on multiple immune cell types in the TME and the secondary lymphoid system.

### HSA-IL21 and PD-1 Blockade Combination Therapy Additively Inhibited Tumor Growth

**FIGURE 2:**
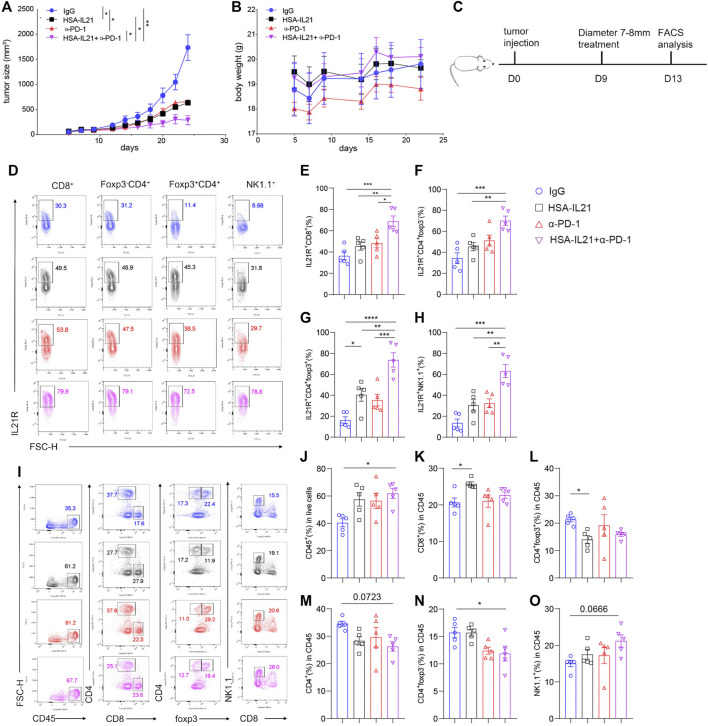
HSA-IL21 and PD-1 blockade combination therapy additively inhibited tumor growth. **(A,B)** MC38 tumor cells (1 × 10^6^) were injected intradermally to B6 mice, monitored tumor volume **(A)** and body weight **(B)** of tumor-bearing mice treated with IgG, HAS-IL21, α-PD-1, HSA-IL21/α-PD-1. Data were presented as mean ± SEM, *n* = 4–5, *p < 0.05, **p < 0.01, ***p < 0.001, ****p < 0.0001, two-way ANOVA test were performed. (C) Graphic schematics of the mouse experiment. MC38 tumor cells (1 × 10^6^) were injected intradermally to B6 mice, 9 days after tumor inoculation, MC38 tumor-bearing mice were treated with IgG, HSA-IL21, α-PD-1, HSA-IL21/α-PD-1.96h later, tumor-infiltrating lymphocytes were analyzed by flow cytometry. **(D)** Representative flow plots of IL21R expression on tumor-infiltrating CD8^+^, Foxp3^-^CD4^+^, Foxp3^+^CD4^+^ T and NK cells. **(E–H)** Quantitative analysis of IL21R expression depicted in **(D)**. Data were presented as mean ± SEM, *n* = 5, *p < 0.05, **p < 0.01, ***p < 0.001, ****p < 0.0001, one-way ANOVA test were performed. **(I)** Representative flow plots showing the percentage of main immune populations. **(J–O)** Quantitative percentage of tumor infiltrating CD45^+^ lymphocytes, percentages of CD8^+^ T, CD4^+^ T cells, Foxp3^+^CD4^+^, Foxp3^-^CD4^+^ T and NK cells. Data were presented as mean ± SEM, *n* = 5, *p < 0.05, **p < 0.01, ***p < 0.001, ****p < 0.0001, one-way ANOVA test was performed.

In order to improve pharmacologic property of IL21, an anti-HSA nanoantibody was fused to IL21. The half-life of IL21 was extended less than 30 min to more than 15 h in the mouse (Zhong et al., 2020). We then tested the efficacy *in vivo* of HSA-IL21 administration alone and in combination with PD-1 blockade. HSA-IL21 alone significantly inhibited the growth of MC38 tumors at comparable levels to PD-1 blockade (Figure 2A). The weight of the mice stayed constant, showing that HSA-IL21 has no apparent toxicity (Figure 2B). Combination therapy completely stunted tumor growth (Figure 2A and Supplementary Figure S2A). Administration of recombinant IL21 at the same dose and frequency did not produce any antitumor effect (Supplementary Figures S2C,D).

To uncover the mechanisms of HSA-IL21/PD-1 blockade combination therapy, we studied the composition and functional states of immune cells in the TME using multi-color flow cytometry. Combination therapy additively increased the expression of IL21R on Foxp3^-^ CD4^+^ T cells, Tregs, CD8^+^ T cells, and NK cells in the TME (Figures 2C–H). HSA-IL21 or PD-1 blockade alone increased the fraction of total immune cells (CD45^+^) out of all cells in the TME, though combination therapy did not cause any further increases (Figures 2I,J). Within the CD45^+^ immune cell compartment, HSA-IL21 treatment increased the fraction of CD8^+^ T cells and decreased the fraction of Treg cells (Figures 2K,L). In contrast, combined therapy decreased the fraction of CD4^+^ T cells (Figures 2M,N). Combination therapy shifted the T cell compartment towards the HSA-IL21 phenotype. The fraction of NK cells was not significantly altered by HSA-IL21 or PD-1 blockade alone. However, combination therapy significantly increased the fraction of NK cells (Figure 2O). These data show that HSA-IL21/PD-1 blockade combination therapy might act via IL21R to increase the immune response in the TME and thereby inhibit tumor growth.

### Combination Therapy Enhances the Effector Function of Tumor-Infiltrating Lymphocytes

**FIGURE 3:**
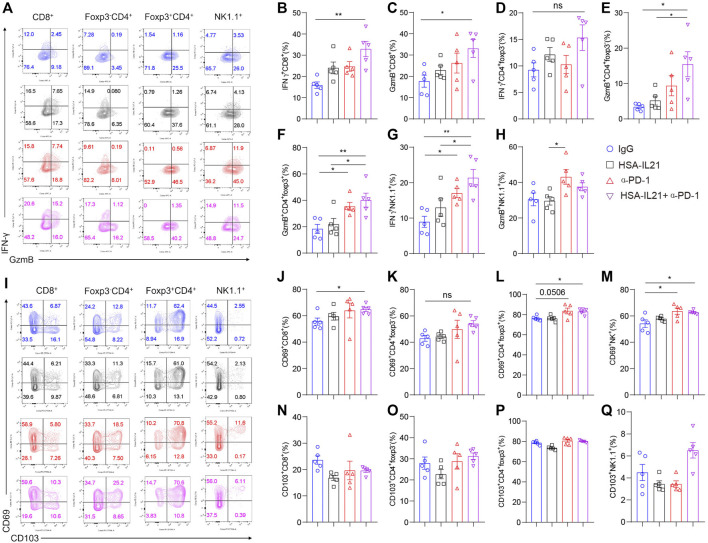
Combination therapy enhances the effector function of tumor-infiltrating lymphocytes. **(A–Q)** MC38 tumor cells (1×10^6^) were injected intradermally to B6 mice, 9 days after tumor inoculation, MC38 tumor-bearing mice were treated with IgG, HSA-IL21, α-PD-1, HSA-IL21/α-PD-1, 96 h later, tumors were resected and analyzed by flow cytometry. (A and I) Representative flow plots showing IFN-γ, GzmB, CD69 and CD103 staining in CD8^+^, Foxp3^-^CD4^+^, Foxp3^+^CD4^+^ and NK cells. **(B–H)** Quantitative percentage of IFN-γ and GzmB expression in CD8^+^, Foxp3^-^CD4^+^, Foxp3^+^CD4^+^ T and NK cells. Data were presented as mean ± SEM, *n* = 5, *p < 0.05, **p < 0.01, ***p < 0.001, ****p < 0.0001, one-way ANOVA test was performed. **(J–Q)** Quantitative percentage of CD69 expression in CD8^+^, Foxp3^-^CD4^+^, Foxp3^+^CD4^+^ T and NK cells. Data were presented as mean ± SEM, *n* = 5, *p < 0.05, **p < 0.01, ***p < 0.001, ****p < 0.0001, one-way ANOVA was performed.

To determine whether combination therapy affects the effector function of tumor-infiltrating lymphocytes (TILs), we examined the effector and activation molecules GzmB, IFN-γ, and CD69 on CD8^+^, Foxp3^-^CD4^+^, Foxp3^+^CD4^+^ T cells and NK cells in the TME at 96 h after treatment by using multi-color flow cytometry (Figures 3A,I). The production of both IFN-γ and GzmB by CD8^+^ T cells and CD4^+^ T cells was additively increased by combination therapy (Figures 3B–H). Combination therapy also additively increased IFN-γ production on NK cells. GzmB production was enhanced by combination therapy or PD-1 blockade, but not HSA-IL21 alone (Figures 3G,H). The expression of CD69, a marker for activated and tissue resident T cells (Radulovic et al., 2013), was additively increased in Foxp3^-^ CD4^+^ T cells, Tregs, CD8^+^ T cells, and NK cells following combination therapy. However, the expression of CD103, another marker for tissue resident T cells (Topham and Reilly, 2018), was unchanged. (Figures 3B,J–Q). Overall, HSA-IL21/PD-1 blockade combination therapy increases TIL effector function.

### Combination Therapy Induces Expression of Immune Checkpoint Molecules on Tumor-Infiltrating Lymphocytes

**FIGURE 4:**
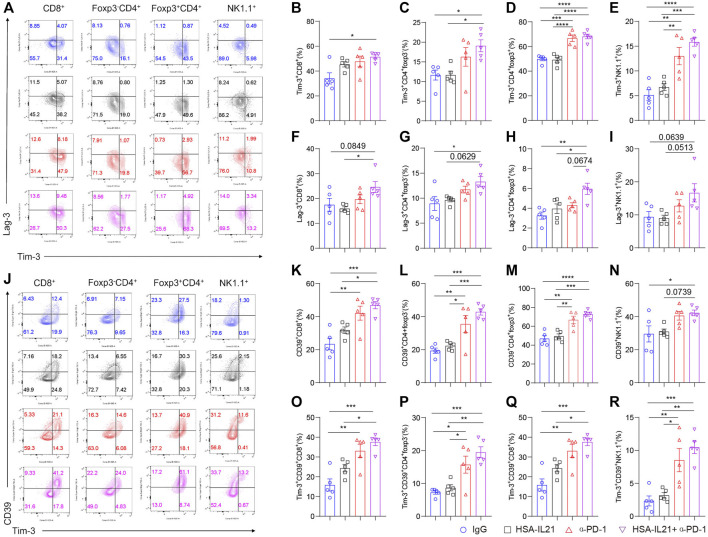
Combination therapy induces expression of immune checkpoint molecules on tumor-infiltrating lymphocytes. **(A–I)** MC38 tumor cells (1 × 10^6^) were injected intradermally to B6 mice, 9 days after tumor inoculation, MC38 tumor-bearing mice were treated with IgG, HSA-IL21, α-PD-1, HSA-IL21/α-PD-1, 96 h later, tumors were resected and analyzed by flow cytometry. **(A)** Representative flow plots showing Tim-3 and Lag-3 staining in CD8^+^, Foxp3^-^CD4^+^, Foxp3^+^CD4^+^ T and NK cells. **(B–I)** Quantitative percentage of Tim-3 and Lag-3 expression in CD8^+^, Foxp3^-^CD4^+^, Foxp3^+^CD4^+^ T and NK cells. Data were presented as mean ± SEM, *n* = 5, *p < 0.05, **p < 0.01, ***p < 0.001, ****p < 0.0001, one-way ANOVA test was performed. **(J)** Representative flow plots showing Tim-3 and CD39 staining in CD8^+^, Foxp3^-^CD4^+^, Foxp3^+^CD4^+^ and NK cells. **(K–R)** Quantitative percentage of CD39 and CD39/Tim-3 double positive expression in CD8^+^, Foxp3^-^CD4^+^, Foxp3^+^CD4^+^ T and NK cells. Data were presented as mean ± SEM, *n* = 5, *p < 0.05, **p < 0.01, ***p < 0.001, ****p < 0.0001, one-way ANOVA test was performed.

Since the expression of immune checkpoint molecules characterizes T and NK cell exhaustion and may limit the efficacy of combination therapy (Wherry, 2011; Wherry and Kurachi, 2015; Yang et al., 2020; Moesta et al., 2020; Bastid et al., 2015; Zhang et al., 2019; Sade-Feldman et al., 2019; Haas and Obenauf, 2019; Sun et al., 2021), we next looked at the expression of checkpoint molecules Tim-3, Lag-3, and CD39 by multi-color flow cytometry. The expression of all three inhibitory receptors was significantly enhanced on Foxp3^-^CD4^+^ T cells, Tregs, CD8^+^ T cells, and NK cells after combination therapy (Figures 4A–N). In addition, the fraction of Tim-3^+^CD39^+^ CD8^+^ T cells was significantly enhanced after combination treatment (Figures 4J,O–R). These results indicate that HSA-IL21/PD-1 blockade combination therapy may drive TILs into a hyperactivated state that is controlled by multiple immune checkpoint molecules (Yang et al., 2020).

### Combination Therapy Promoted DC1 and M1 Cells and Decreased DC2 and M2 Cells in the TME

**FIGURE 5:**
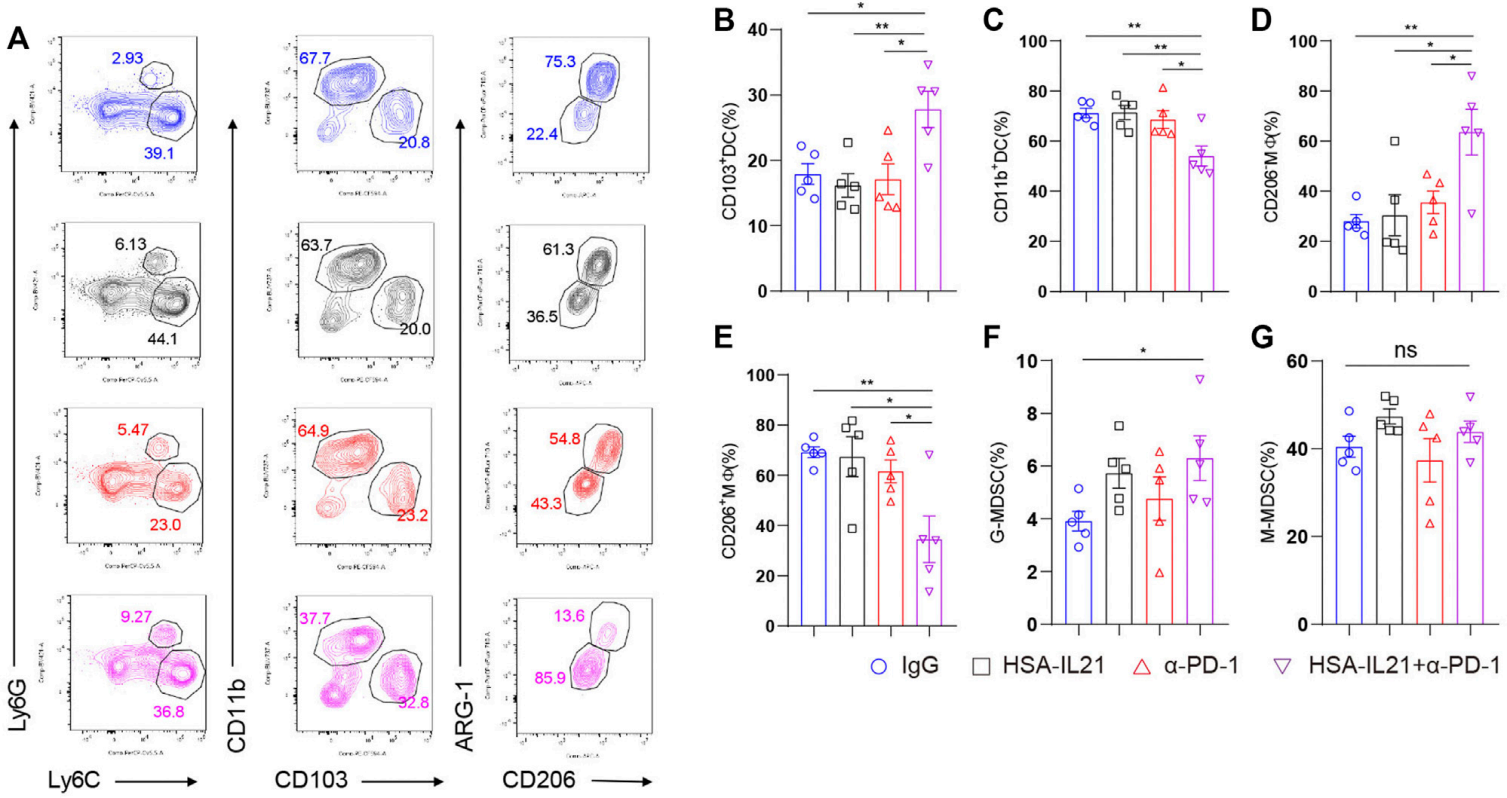
Combination therapy induced myeloid cells that promote the CD8^+^ T cell-mediated immune response against cancer. **(A–G)** MC38 tumor cells (1 × 10^6^) were injected intradermally to B6 mice, 9 days after tumor inoculation, MC38 tumor-bearing mice were treated with IgG, HSA-IL21, α-PD-1, HSA-IL21/α-PD-1, 96h later, tumors were resected and analyzed by flow cytometry. Representative flow plots showing the percentage of myeloid populations. **(B–G)** Quantitative percentage of tumor infiltrating dendritic cells, macrophage, MDSC. Data were presented as mean ± SEM, *n* = 5, *p < 0.05, **p < 0.01, ***p < 0.001, ****p < 0.0001, one-way ANOVA test was performed.

We next investigated whether combination therapy affected the myeloid compartment of the TME using multi-color flow cytometry. We found an increase in the fraction of DC1 and M1 macrophages in the immune compartment following combination therapy (Figures 5A–E). In contrast, the fraction of M2 macrophages and DC2 was decreased. These results are consistent with the observed increases in the CD8^+^ T cell-mediated immune response. Interestingly, we found that combination therapy increases the fraction of immunosuppressive MDSCs in the TME (Figures 5A,F,G). These data show that the myeloid compartment mainly promotes the anti-tumor lymphocyte response following combination therapy, but that some immunosuppression also occurs via MDSCs.

### Combination Therapy Promotes the Tumor-antigen-specific T Cell Response in Peripheral Lymphoid Organs

**FIGURE 6:**
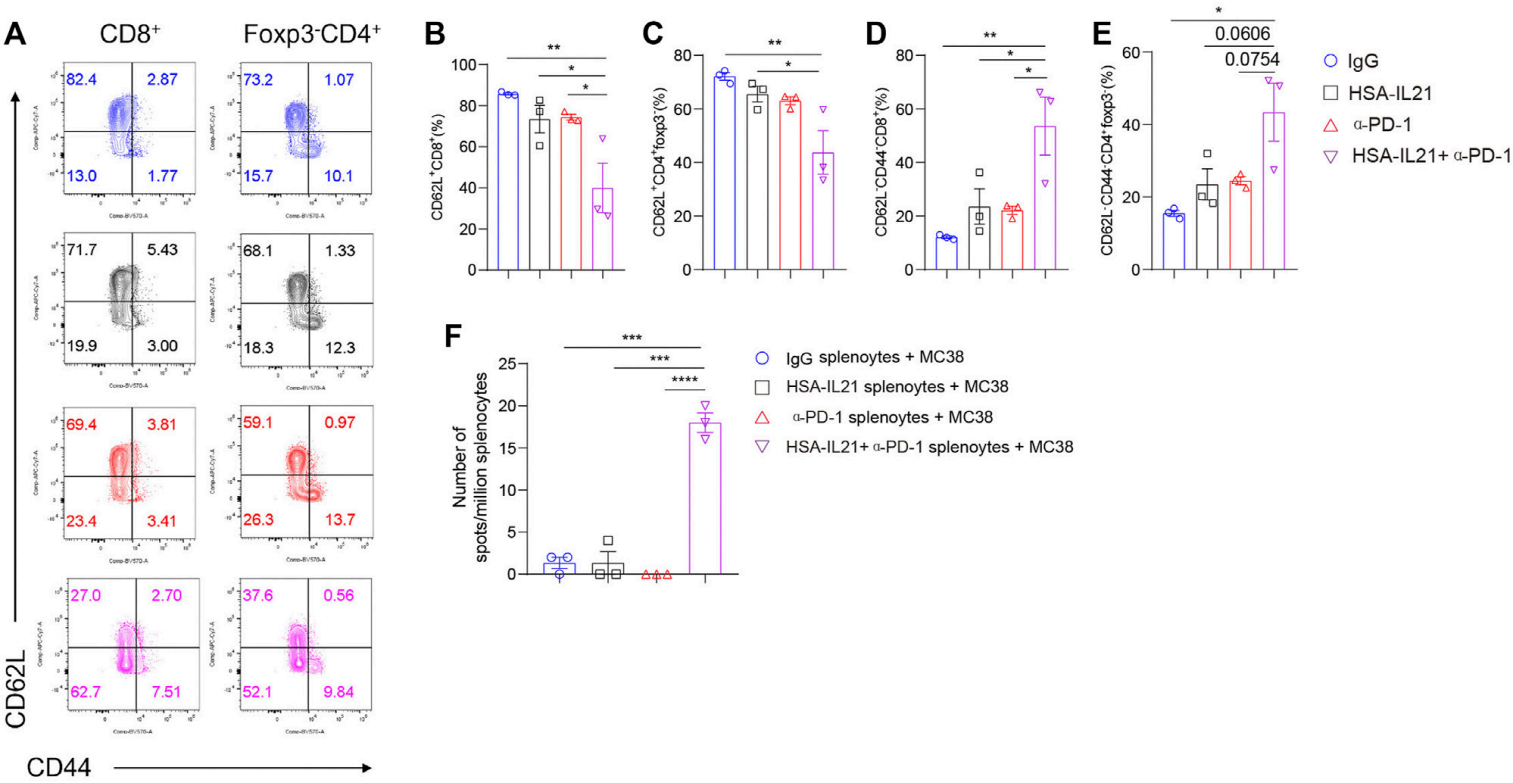
Combination therapy promotes the tumor-antigen-specific T cell response activation in peripheral lymphoid organs. **(A–F)** MC38 tumor cells (1 × 10^6^) were injected intradermally to B6 mice, 9 days after tumor inoculation, MC38 tumor-bearing mice were treated with IgG, HSA-IL21, α-PD-1, HSA-IL21/α-PD-1, 96 h later, immune cells from spleen were analyzed by flow cytometry. Splenocytes were applied to IFN-γ ELISpot assay. **(A)** Representative flow plots showing CD62L and CD44 staining on CD8^+^ and Foxp3^-^CD4^+^T cells in spleen. Quantitative expression of CD44, CD62L expression on T cells gated on CD8^+^ **(B–D)** and Foxp3^-^CD4^+^T cells **(E)** in spleen. Data were presented as mean ± SEM, *n* = 3, *p < 0.05, **p < 0.01, ***p < 0.001, one-way ANOVA test was performed. **(F)** Numbers of tumor-antigen specific IFN-γ producers in the splenocytes. Data were presented as mean ± SEM, n = 3, *p < 0.05, **p < 0.01, ***p < 0.001, one-way ANOVA test was performed.

We also examined T cells in the spleen to determine changes in the peripheral lymphoid organs following combination therapy. We found no difference in the percentages of Foxp3^-^ CD4^+^ T cells and CD8^+^ T cells. However, combination therapy additively decreased the fraction of naïve CD62L^+^ CD8^+^ and CD62L^+^ Foxp3^-^ CD4^+^T cells (Figures 6A–C). The fraction of CD62L^-^CD44^-^CD8^+^ or CD62L^-^CD44^-^Foxp3^-^CD4^+^ T cells were increased in the combination therapy group (Figures 6A,D,E). There data suggest that the combined treatment with HSA-IL21 and PD-1 mAbs resulted in a systemic decrease in naive T cells. In order to further determine tumor-antigen-specific T cells, we performed the enzyme-linked immunosorbent spot (ELISpot) assay. We found that tumor antigen-specific effector T cells in the spleen were significantly increased upon combination therapy (Figure 6F). These data show that HSA-IL21/PD-1 mAbs combination therapy leads to both systemic T cell activation and an increase in the number of tumor-antigen-specific T cells in the peripheral lymphoid organs.

### Combination Therapy Sustains Anti-Tumor Immune Responses in TME and Periphery

**FIGURE 7:**
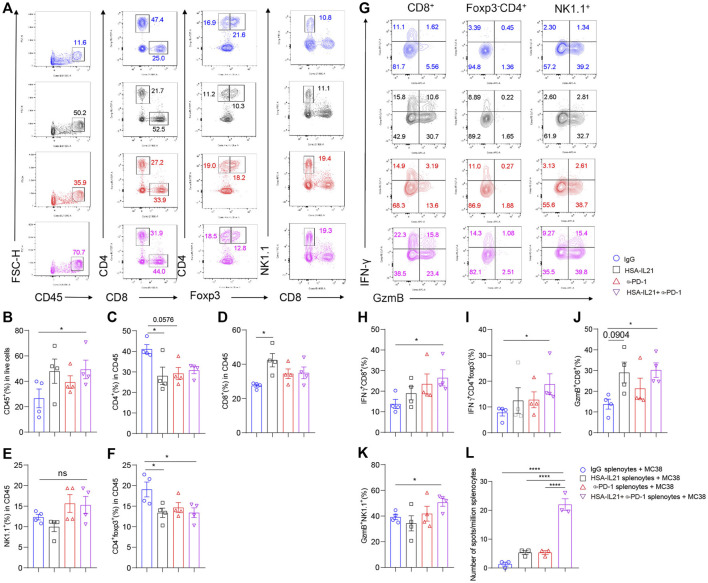
Combination therapy sustains anti-tumor immune responses in TME and periphery. **(A–L)** MC38 tumor cells (1 × 10^6^) were injected intradermally to B6 mice, 5 days after tumor inoculation, MC38 tumor-bearing mice were treated with IgG, HSA-IL21, α-PD-1, HSA-IL21/α-PD-1 for total 4 times, after last treatment 48h, tumors were resected and analyzed by flow cytometry (*n* = 4). **(A)** Representative flow plots showing the percentage of main immune populations. **(B–F)** Quantitative percentage of tumor infiltrating CD45^+^ lymphocytes, percentages of CD8^+^, CD4^+^, Foxp3^-^CD4^+^, Foxp3^+^CD4^+^T cells and NK cells. Data were presented as mean ± SEM, *n* = 4, *p < 0.05, **p < 0.01, ***p < 0.001, ****p < 0.0001, one-way ANOVA test was performed. **(G)** Representative flow plots showing IFN-γ and GzmB staining in CD8^+^, Foxp3^-^CD4^+^ T cells and NK cells. **(H–K)** Quantitative percentage of IFN-γ and GzmB expression in CD8^+^, Foxp3^-^CD4^+^T cells and NK cells. Data were presented as mean ± SEM, n = 4, *p < 0.05, **p < 0.01, ***p < 0.001, ****p < 0.0001, one-way ANOVA test were performed. **(L)** Numbers of tumor-antigen specific IFN-γ producers in the splenocytes. Data were presented as mean ± SEM, *n* = 3, *p < 0.05, **p < 0.01, ***p < 0.001, ****p < 0.0001, one-way ANOVA test was performed.

Given the strong anti-tumor immune response induced by combination therapy, we decided to test whether the therapy could sustain these responses overtime. We repeatedly administered of the combination therapy over four times at a 4-day interval, and performed multi-color flow cytometry of the TME (Figure 7). We found moderate increases in the fraction of total immune cells in the TME (Figures 7A,B). Within the immune compartment, we found a significant increase in the fraction of CD8^+^ T cells in the TME of the IL21 treatment group (Figures 7A,B,D,E). The fraction of CD4^+^ Foxp3^-^ T cells and Tregs significantly decreased in all treatment groups (Figures 7A,C,F). We also found that combination therapy additively increased the fraction of IFN-γ^+^ CD4^+^ T cells, IFN-γ^+^ CD8^+^ T cells, GzmB^+^ CD8^+^ T cells, and GzmB^+^ NK cells (Figures 7G–K). The number of tumor antigen-specific T cells in the spleen were also increased upon treatment with combination therapy (Figure 7L). Collectively, these data demonstrate that combination therapy continually promotes the type 1 immune response over time and IL21 in particular greatly increases the tumor-antigen specific T cell in the periphery.

### HSA-IL21 Combined With Tim-3, Lag-3, and PD-1 Blockade Additively Inhibits Tumor Growth

**FIGURE 8:**
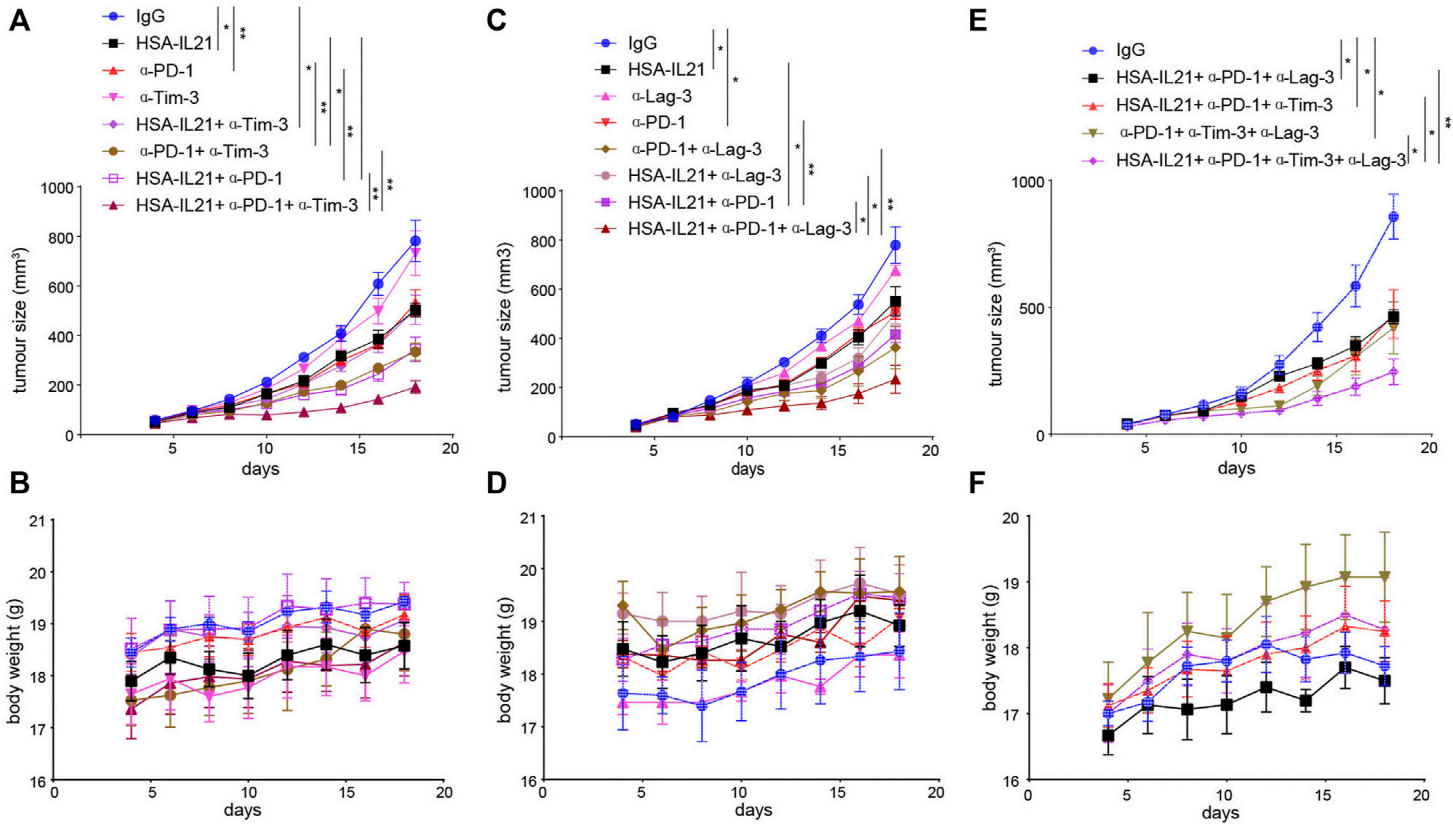
HSA-IL21 combined with Tim-3, Lag-3, and PD-1 blockade additively inhibits tumor growth. **(A,B)** MC38 tumor cells (1×10^6^) were injected intradermally to B6 mice, 5 days after tumor inoculation, MC38 tumor-bearing mice were treated with IgG, HSA-IL21, α-PD-1, α-Tim-3, HSA-IL21/α-PD-1, HSA-IL21/α-Tim-3, α-PD-1/α-Tim-3, HSA-IL21/α-PD-1/α-Tim-3, for total 4 times. Tumor size **(A)** and body weight **(B)** were measured every 2 days. (*n* = 4–5). Data were presented as mean ± SEM, *n* = 5, *p < 0.05, **p < 0.01, ***p < 0.001, ****p < 0.0001, two-way ANOVA test was performed. **(C,D)** MC38 tumor cells (1×10^6^) were injected intradermally to B6 mice, 5 days after tumor inoculation, MC38 tumor-bearing mice were treated with IgG, HSA-IL21, α-PD-1, α-Lag-3, HSA-IL21/α-PD-1, HSA-IL21/α-Lag-3, α-PD-1/α-Lag-3, HSA-IL21/α-PD-1/α-Lag-3, for total 4 times. Tumor size(C) and body weight(D) were measured every 2 days. (*n* = 3–5). Data were presented as mean ± SEM, *n* = 5, *p < 0.05, **p < 0.01, ***p < 0.001, ****p < 0.0001, two-way ANOVA test were performed. (E–F) MC38 tumor cells (1 × 10^6^) were injected intradermally to B6 mice, 5 days after tumor inoculation, MC38 tumor-bearing mice were treated with IgG, HSA-IL21/α-PD-1/α-Tim-3, HSA-IL21/α-PD-1/α-Lag-3, α-PD-1/α-Tim-3/α-Lag-3, HSA-IL21/α-PD-1/α-Tim-3/α-Lag-3, for total 4 times. Tumor size **(E)** and body weight **(F)** were measured every 2 days. (*n* = 3–5). Data were presented as mean ± SEM, *n* = 5, *p < 0.05, **p < 0.01, ***p < 0.001, ****p < 0.0001, two-way ANOVA test was performed.

Given that HSA-IL21/PD-1 blockade combination therapy increases the expression of checkpoint molecules Tim-3 and Lag-3 expression on TILs, we next determined whether the efficacy the therapy could be improved by combination with ICB therapies targeting Tim-3 and Lag-3. Our results indicate that both HSA-IL21/PD-1/Tim-3 or Lag-3 blockade triple combinations produced a greater antitumor effect without apparent toxicity (Figures 8A–D). Moreover, HSA-IL21/PD-1/Tim-3/Lag-3 blockade quadruple therapy produced an even greater antitumor effect, again without apparent toxicity (Figures 8E,F and Supplementary Figures S3A–C). Our study shows that HSA-IL21 can be combined with multiple checkpoint inhibitors to improve current cancer immunotherapies.

## Discussion

Our work demonstrates that the half-life-extended HSA-IL21 retains the antitumor effect of WT IL21 and produces superior efficacy when combined with PD-1 blockade *in vivo*. The cellular mechanisms behind the additive effect of combination therapy involve increases in the fraction and effector functions of CD8, Th1 and NK cells, decreases in the fraction of Treg cells, increases in the fraction of DC1 and M1 macrophages, and increases in the number of tumor-antigen-specific T cells in the peripheral lymphoid organs. The additive effect provided by combination therapy shows that IL21 as a cancer immunotherapeutic is limited by the immune checkpoint molecule PD-1. We found that the effect of IL21 is also limited by multiple checkpoint molecules, including Tim-3 and Lag-3—combination therapies involving blockade of these molecules further improved therapeutic efficacy without causing severe toxicity. We found that the effect of IL21 is further limited by MDSCs. Our findings chart pathways for further improvement of IL21-based therapy.

We found that IL21 and PD-1 blockade alone or in combination act on T and NK cells. Combination therapy additively increases IL21R expression on T cells and NK cells in the TME. This finding suggests that IL21R might be an important molecular hub that integrates the signaling pathways of both IL21 and PD-1 blockade on TILs. We also showed that combination therapy synergistically increases the production of GzmB and IFN-γ by TILs. This is likely due to direct regulation of these genes by IL21 because a previous study showed that GzmB is an IL21 target gene (Shourian et al., 2019). Consistent with our conclusions, this study found that IL21 sustains the cytotoxic functions of CD8 T cells and increases their cytokine secretion capacities (Shourian et al., 2019). It has been shown that IL21 increases the number of central memory T cells and T memory stem cells *in vitro* (Kasaian et al., 2002; Brady et al., 2004; Zeng et al., 2005; Zhang et al., 2005; Klebanoff et al., 2011; Wölfl et al., 2011; Chen et al., 2018). Here, we found that IL21 increases the number of tumor-antigen-specific T cells in the spleen *in vivo*. These results show that IL21/PD-1 blockade combination can increase both effector function as well as the number of tumor antigen-specific T cells.

IL21/PD-1 blockade combination therapy activates TILs, but also induces the expression of immune checkpoint molecules Lag-3, Tim-3 and CD39. The strong stimulation provided by IL21 and PD-1 blockade on effector T cells may result in hyperactivation, a state that is characterized by the expression of multiple immune inhibitory receptors. These receptors prevent immune-mediated pathology, but limits the antitumor activity (Alvarez-Fernández et al., 2016; Haas and Obenauf, 2019; Sade-Feldman et al., 2019; Sun et al., 2021). We showed that triple combination therapy with Tim-3 or Lag-3, and quadruple therapy, further increases the efficacy of double therapy. Combination therapies should be tested in the clinic to further improve patient outcomes.

We showed that HSA-IL21/PD-1 blockade combination therapy increases the fraction of DC1 cells in the TME. Consistent with this finding, we observed an increase in the fraction and function of CD8^+^ T cells. Our results on DC1 contrast previous studies showing that IL21 has a potent inhibitory effect on DCs(Brandt et al., 2003; Wan et al., 2013). One study showed that addition of IL21 during generation of mouse bone marrow-derived DCs (BMDCs) reduces the expression of major histocompatibility complex II (MHCII) and the ability to induce antigen-specific CD4^+^ T cell proliferation (Brandt et al., 2003). IL21 added during lipopolysaccharide (LPS) stimulation inhibits DC activation and maturation, as well as the production of proinflammatory cytokines IL-1β, IL-12, IL-6, and tumor necrosis factor α (TNF-α). Another study showed that IL21 induces apoptosis of splenic conventional dendritic cells (cDCs) via induction of Bim (Wan et al., 2013). Consistent with these results, we also found that combination therapy decreases the fraction of DC2s in the TME. IL21 might act on DC2s in order to decrease the fraction of CD4^+^ Foxp3^-^ T cells or Tregs in the TME. Our findings suggest that IL21 differentially acts on DC1s vs DC2s to promote the CD8^+^ T cell-mediated anti-tumor immune response.

Consistent with activation of CD8^+^, Th1 cells, and NK cells, we found that combination therapy increased the fraction of M1 macrophages and decreased the fraction of M2 macrophages in the TME. Again, we found increased the effector function of CD4^+^ Foxp3^-^ T cells, CD8^+^ T cells, and NK cells. Combination therapy primarily shifts the immune response to a type 1 phenotype that is anti-tumor. However, we also observed increases in the fraction of immunosuppressive mMDSCs and nMDSCs out of all immune cells. The mechanism by which IL21 acts on MDSCs requires further work to be uncovered. Nonetheless, our findings highlight the opportunity to combine IL21 with immunotherapies that target MDSCs.

Recombinant IL21 as a single agent has been evaluated in clinical trials with encouraging efficacy. However, there are no published results about combination therapies using both IL21 and ICB in human cancer patients. The requirement for frequent dosing might be an obstacle to the success of IL21-based therapies in cancer patients. Our study demonstrates that the half-life extended HSA-IL21 has potential to be combined with existing ICB therapies as well as multiple additional checkpoint inhibitors in the clinic.

## Materials and Methods

### Mouse

C57BL/6J and BALB/c mice were purchased from The Jackson Laboratory and housed in the specific pathogen-free animal facility of University of Pittsburgh School of Medicine. All mice experiments have been approved by Institutional Animal Care and Use Committee of University of Pittsburgh.

### Cell Culture and Tumor Model

MC38 tumor cell line was cultured in complete DMEM medium plus with 10% fetal bovine serum (FBS) and 1% penicillin-streptomycin (P.S). CT26 cancer cells are transfected with plasmid coding MSLN ORF, and selected by surface marker MSLN using fluorescenec-activated cell sorting (FACS). CT26-MSLN tumor cell line was cultured in complete DMEM medium plus with 10% fetal bovine serum (FBS) and 1% penicillin-streptomycin (P.S). For MC38 tumor model, C57BL/6 mice were injected with 1 million cells intradermally (i.d.). For CT26-MSLN tumor model, 1 million cells were injected intradermally (i.d.) into BALB/c mice. MC38 and CT26 bearing mice were randomized into four treatment cohorts: control IgG, HSA-IL21, α-PD-1 (clone J43, BioXCell) or HSA-IL21/α-PD-1. HSA-IL21 was injected by intraperitoneally (i.p.) 25 μg per mouse, α-PD-1 were injected by intraperitoneally (i.p.) 200 μg per mouse. All mice were administered on the 5th day after tumor inoculation. Tumor sizes were monitored every 2–3 days, and the tumor volume was calculated as L× W^2^/2.

### Reagents and Antibodies

HSA-IL21 (the half-life extended IL21 was kindly provided by Anwita Biosciences, CA, USA), PD-1mAb (clone:J43), hamster IgG were purchased from Bioxcell company (catalog no. BE0091) for tumor therapy. For Flow cytometry, CD45 (clone: 30-F11), CD4 (clone: GK1.5), CD8 (clone: 53–6.7), NK1.1 (clone: PK136), B220 (clone: RA3-6B2), Foxp3 (MF-14), PD-1(29F.1A12), Tim-3 (clone: RMT3-23), Lag-3 (clone: C9B7W), CD39 (clone: 24DMS1), CD62L (clone: MEL-14), CD44 (clone: IM7), CD103 (clone: M290), CD69 (clone: H1.2F3), IFN-γ (clone: XMG1.2), GzmB (clone: QA16A02), CD11b (clone: M1/70), Ly6C (clone: HK1.4), Ly6G (clone: 1A8), Gr-1 (clone: RB6-8C5), CD24 (clone: M1/69), F4/80 (clone: BM8), CD11C (clone: N418), CD206 (clone: MR6F3), Arginase 1 (clone: A1exF5) were purchased from Biolegend ebioscience or BD Bioscience. Zombie NIR dye was purchased from Biolegend.

### Processing of Tissues and Flow Cytometry

Mice were sacrificed, TDLN, spleen and tumors were removed. We placed the spleens and lymph nodes between the frosted surfaces of two glass slides and applied force to disrupt these organs to release immune cells, spleen was need to treated with ACK lysis buffer to remove red blood cells. Single-cell suspensions were filtered through a 40-μm cell strainer, washed, and resuspended in 1%FBS HBS for analysis. Tumor were cut into small pieces, digested in serum free RPMI with 0.25 mg/ml Liberase TL (Roche) and 0.33 mg/ml Dnase 1 (Sigma) in 37° for 30 min. Single-cell suspensions were filtered through a 40-μm cell strainer, washed, and resuspended in 1%FBS HBS for staining. For IFN-γ or Granzyme B staining, tumor cells were stimulated with leukocyte activation cocktail (Biolegend) for 6 h, then stained surface marker and intracellular markers. by the standard staining protocol described before (Chen et al., 2020). Flow cytometry analysis were applied to LSRII or Aurora (Cytek Biosciences) and analyzed by using Flowjo software (BD).

### IFN-γ ELISpot Assay

15 μg/ml capture antibody anti-IFN-γ (clone:AN18, MabTech) was coated and incubated overnight at 4°. On the other day, 5*105 splenocytes were co-cultured with 5*104, 200Gy irradiated MC38 tumor cells at 37° incubator. Forty-8 hours later, Wash the plate 5 times with wash buffer (PBS/0.05% Tween 20) and incubated with 1.5 μg/ml biotinylated secondary antibody (clone: R4-6A2-biotin, MabTech) for 1 h, and then washed and developed with VECTASTAIN Elite ABC HRP Kit (Vector Labs) and incubated with AEC Peroxidase (HRP) Substrate Kit (Vector Labs). The plate was read and counted using the ImmunoSpot Analyzer (Cellular Technology).

### Single-Cell RNA-Seq Data Processing

Zhang et al. (2020) scRNA-seq data of MC38 tumor downloaded from ENA website (ArrayExpress: E-MTAB-8832) was aligned and quantified using the Cellranger Software (Version 4.0.0) against the mm10 mouse reference genome. The preliminary filtered data generated from Cellranger were used for a Seurat object created by the R package Seurat (Version 3.2.3). Doublets were removed by DoubletFinder package. Further quality control was applied to cells based on four metrics step by step, including the total UMI count, number of detected genes and proportion of mitochondrial gene count per cell, and proportion of ribosomal gene count per cell. Specifically, cells with more than 50,000 UMI count and 10% mitochondrial gene count were filtered, as well as cells with more than 50% ribosomal gene count.

### Integration of Multiple scRNA-Seq, Dimension Reduction and Unsupervised Clustering

Single cell data were processed for dimension reduction and unsupervised clustering by following the workflow in Seurat. In brief, 2,000 highly-variable genes were selected for downstream analysis by using FindVariableFeatures function with parameter “nfeatures = 2000.” Subsequently, IntegrateData function was used to integrate data and create a new matrix with 3,000 features, in which potential batch effect was regressed out. To reduce the dimensionality of the scRNA-seq dataset, principal component analysis (PCA) was performed on an scaled integrated data matrix. With ElbowPlot function of Seurat, top 40 PCs were used to perform the downstream analysis. The main cell clusters were identified with the FindClusters function offered by Seurat, with resolution set as default (res = 0.2). And then they were visualized with 2D UMAP plots. Conventional markers described in a previous study were used to categorize every cell into a known biological cell type.

### Statistical Analysis

We used the one-way ANOVA test for comparisons between different treatment groups. Two-way ANOVA was used for comparing tumor growth curves. Statistical analyses were performed with Graphpad Prism.

## Data Availability

The original contributions presented in the study are included in the article/Supplementary Material, further inquiries can be directed to the corresponding authors.

## Supplementary Material

Supplementary Figure S1
**Characterization of IL21R+ peripheral immune cells.** **(A)** Representative flow cytometry gating strategy for identification of immune cell population. (Foxp3-CD4+: CD45+CD4+Foxp3-, Foxp3+CD4+: CD45+CD4+Foxp3+, CD8+: CD45+CD8+, NK cell: CD45+CD8-CD4-NK1.1+, B cells: CD45+CD8-CD4-B220+, CD103+DC: CD45+Gr1-MHCII+CD24+CD103+, CD103-DC: CD45+Gr1-MHCII+CD24+CD103-CD11b+, MDSC: CD45+CD11b+Gr1+, CD206-Mφ: CD45+Gr1-MHCII+F4/80+CD206-Arg-1-, CD206+Mφ: CD45+Gr1-MHCII+F4/80+CD206+Arg-1+, M-MDSC: CD45+CD11b+Ly6c+, G-MDSC: CD45+CD11b+Ly6G+). **(B,C)** Representative flow plots showing IL21R staining on CD8+, CD4+, Foxp3-CD4+, Foxp3+CD4+T cells and NK cells in TDLN **(B)** and spleen **(C)**.

Supplementary Figure S2
**Administration of HSA-IL21 and PD-1 mAbs additively inhibited tumor growth.** **(A)** CT26-MSLN tumor cells (1×10^6^) were injected intradermally to BALB/c mice, monitored tumor volume of tumorbearing mice treated with IgG, IL21, HSA-IL21, α-PD-1, HSA-IL21/α-PD-1. **(B)** Individual tumor curves of mice depicted in Figure S2A. Data were presented as mean±SEM, n=5, *p<0.05, **p<0.01, ***p<0.001, ****p<0.0001, two-way ANOVA test were performed. **(C,D)** MC38 tumor cells (1×10^6^) were injected intradermally to B6 mice, monitored tumor volume **(C)** and individual tumor curves **(D)** of tumor-bearing mice treated with IgG, rIL21, HAS-IL21. Data were presented as mean±SEM, n=5, *p<0.05, **p<0.01, ***p<0.001, ****p<0.0001, twoway ANOVA test were performed.

Supplementary Figure S3
**Administration with Tim-3 or Lag-3, PD-1 mAbs and HSA-IL21 additively inhibited the tumor growth.** **(A–C)** Individual tumor curves of mice depicted in Figures 8A,C,E.
