# Supplementary figures and images for: The Half-Life-Extended IL21 can Be Combined With Multiple Checkpoint Inhibitors for Tumor Immunotherapy
Source: Front Cell Dev Biol. 2021 Nov 15;9:779865. doi: 10.3389/fcell.2021.779865 (PMC8634682; doi:10.3389/fcell.2021.779865)

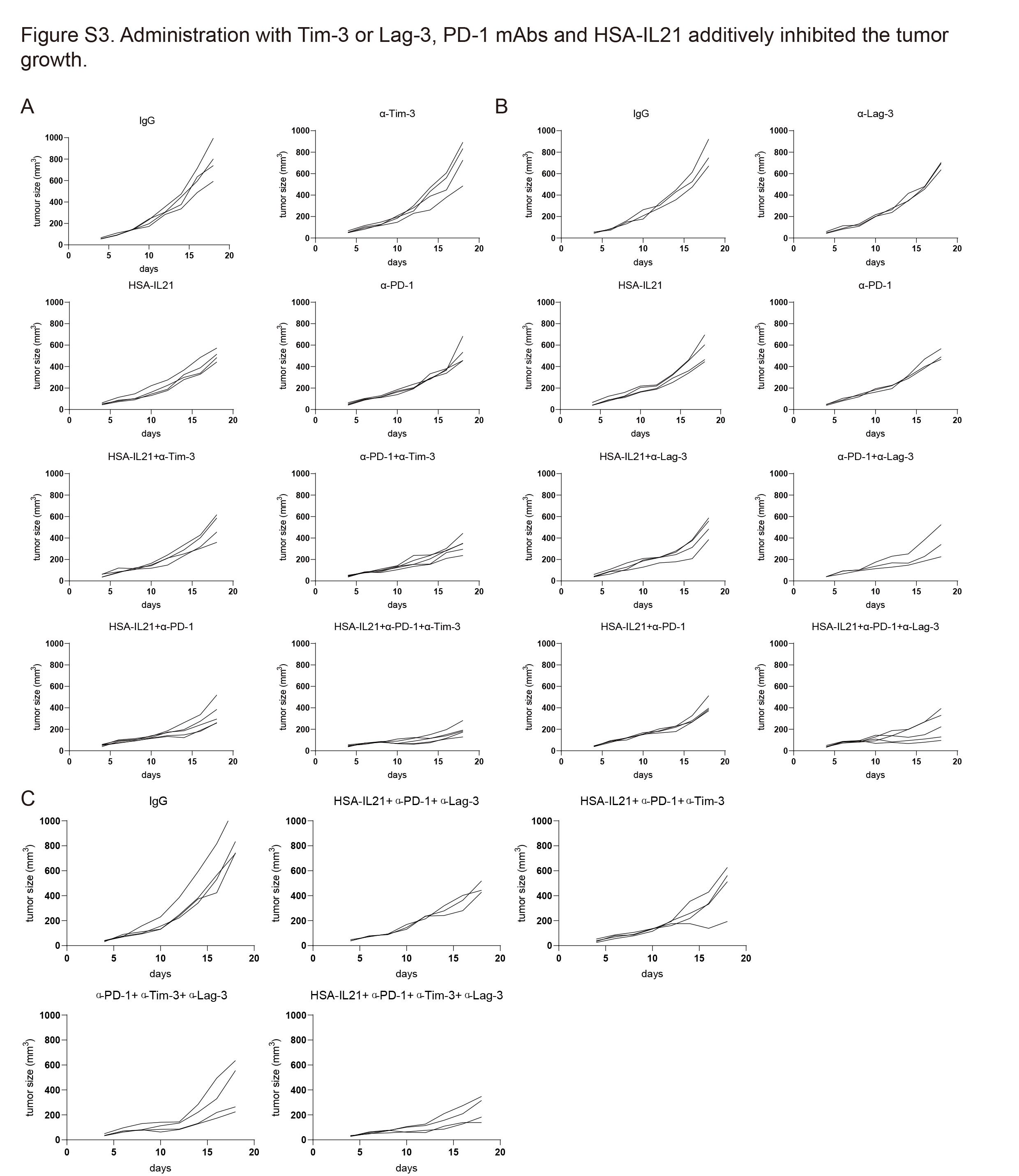

Supplement: Supplementary file 1 [file Image3.jpeg]

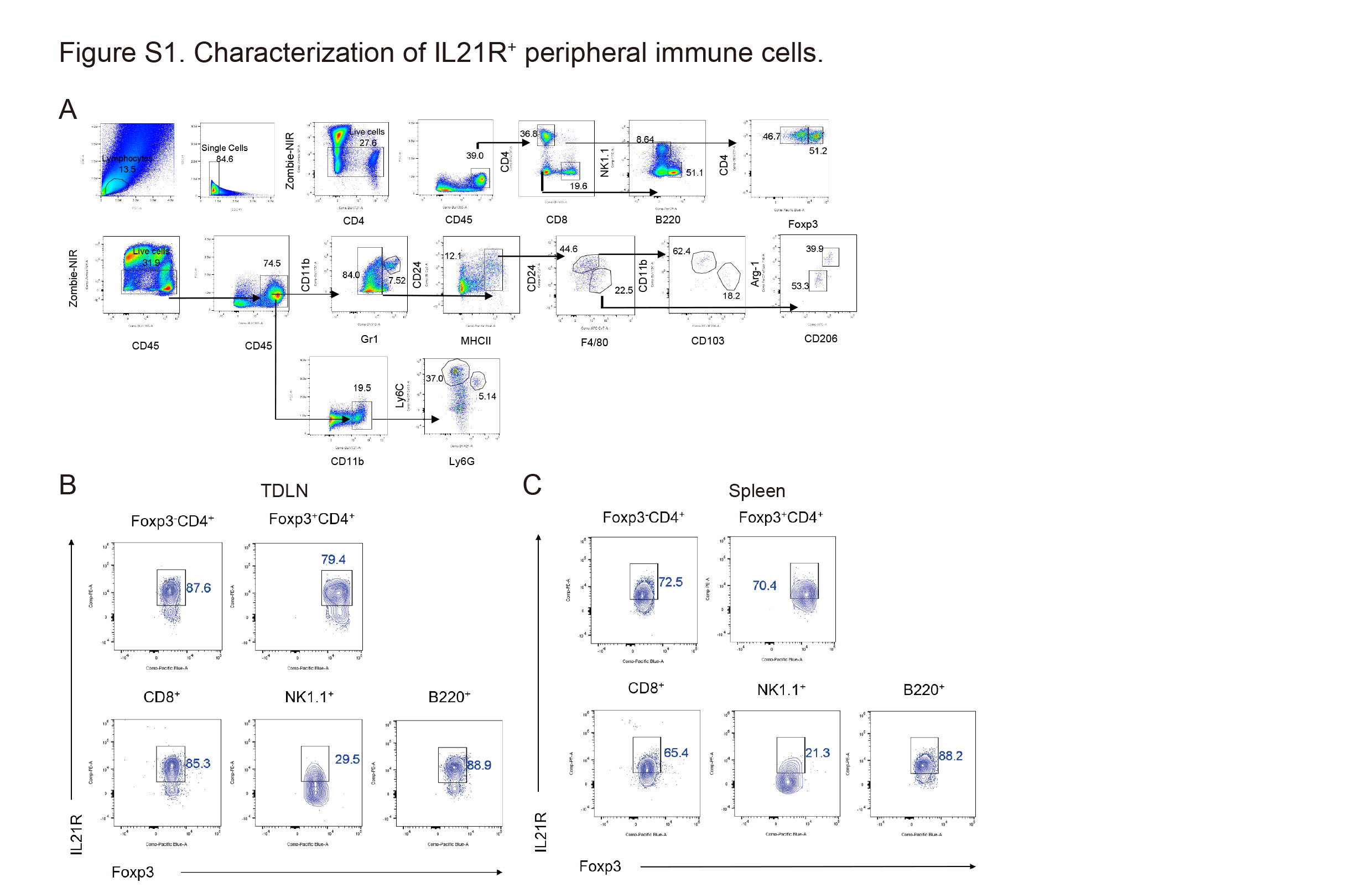

Supplement: Supplementary file 2 [file Image1.jpeg]

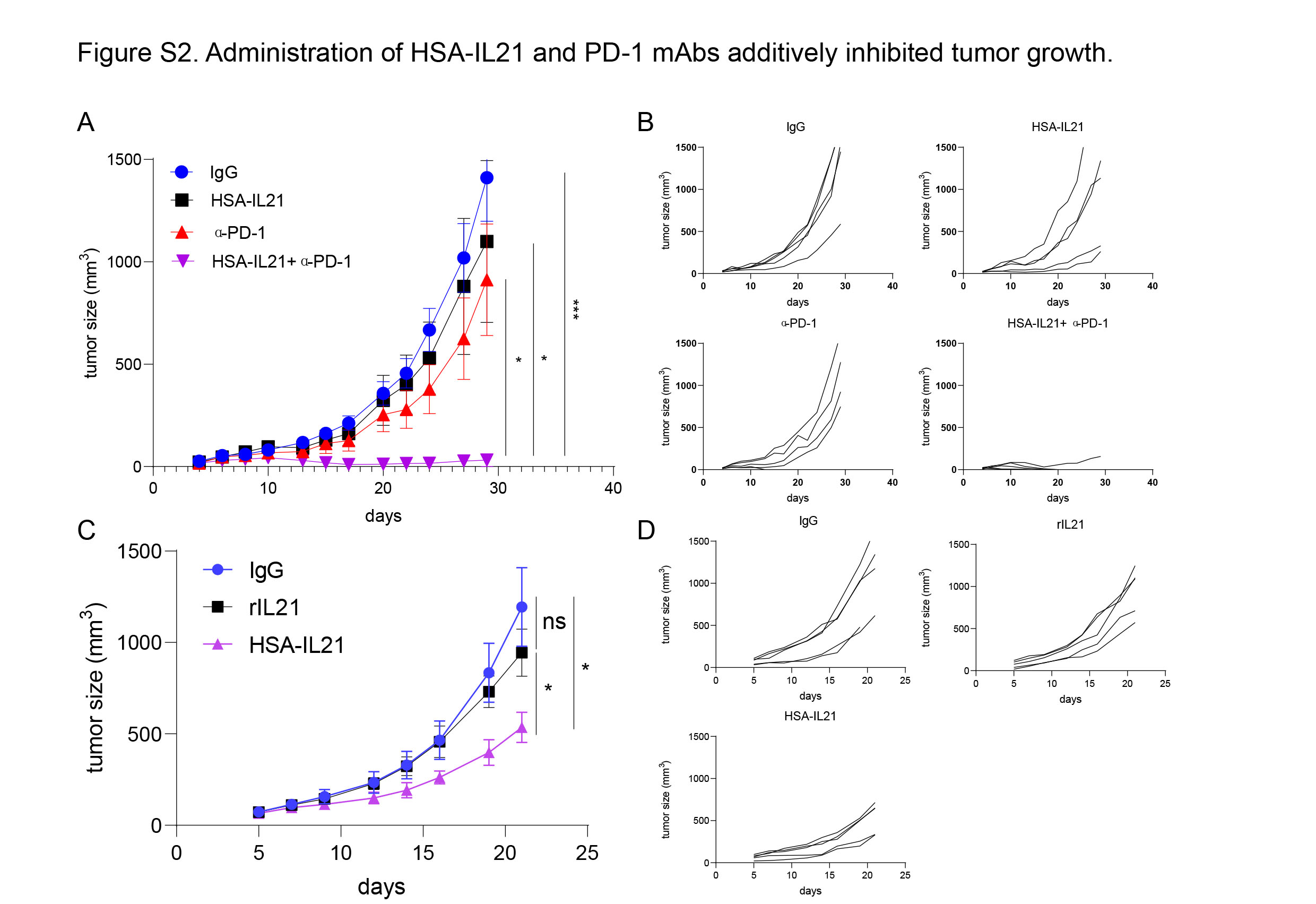

Supplement: Supplementary file 3 [file Image2.jpeg]
